# Missed preoperative nursing care in dentistry

**DOI:** 10.3389/froh.2025.1603814

**Published:** 2025-09-02

**Authors:** Chukwuemeka L. Anyikwa, Peter A. Brennan, Chukwuebuka E. Ogwo

**Affiliations:** 1 College of Medicine, University of Nigeria, Enugu, Nigeria; 2 Maxillofacial Unit, Queen Alexandra Hospital, Portsmouth, United Kingdom; 3 Harvard School of Dental Medicine, Boston, MA, United States

**Keywords:** missed nursing care, pre-operative dentistry, dental nursing care, oral health policy, oral health promotion

## Abstract

Missed preoperative nursing care in dentistry is a critical but overlooked public health issue in many low-resource settings worldwide, including regions across Africa, Southeast Asia, and Latin America. In numerous low and middle-income countries, dental emergencies are managed almost exclusively by dentists, with minimal structured nursing support. This gap in care contributes to delayed interventions, preventable morbidity and mortality, and rising healthcare costs. A systemic approach is urgently needed to integrate nursing into dental emergency management. This includes expanding nursing education to cover oral health, developing standardized nursing protocols for dental infections and fostering multidisciplinary collaboration between the nursing and dental professionals. While successful models from high-income countries offer promising frameworks, their adaptation requires careful consideration of local health system capacities and cultural contexts. Although empirical data on the economic and clinical impact of missed dental nursing care remain limited, addressing this gap is a pressing public health priority. Doing so offers a cost-effective opportunity to strengthen healthcare infrastructure and improve outcomes for vulnerable populations in underserved regions.

## Introduction

Oral health is an essential component of overall health and well-being, yet it remains a neglected priority in many healthcare systems, particularly in low-resource settings across sub-Saharan Africa, Southeast Asia and parts of Latin America (1, 2). Despite the increasing global burden of dental diseases, the integration of nursing care into dental health has received limited policy attention and operational support (3). In regions where surgical dental services are often limited to urban centers, the absence of nursing roles in the perioperative dental pathway is not only a workforce issue but a patient safety concern.

In this context, missed preoperative nursing care in dentistry refers to the omission, delay or incomplete performance of essential nursing interventions prior to dental procedures, particularly those involving infection control or emergency management (4, 5). These tasks may include obtaining baseline vitals, performing risk assessments for systemic spread of infection, identifying early warning signs such as trismus, dysphagia, or facial swelling, and initiating timely referrals. When such care is missed, patients face a heightened risk of developing serious complications, including deep neck infections and sepsis. For instance, in rural Nigeria and northern India, studies have documented cases of odontogenic infections progressing to life-threatening stages due to delays in assessment and referral, situations in which trained nurses could have played a critical triage or monitoring role (6, 7).

Unlike in other medical fields where nurses often serve as frontline providers in surgical and emergency settings, their involvement in dental care appears to be limited. This is largely due to systemic gaps: general nursing curricula in countries such as Eritrea and Nigeria typically lack any oral health modules, and specialized dental nursing programs are either scarce or nonexistent (8, 9). For example, a 2020 survey in Eritrea suggested that 54% of nurses reported a lack of knowledge, and 73.5% noted the absence of guidelines to direct their dental practice (8). Without structured education and protocols, nurses may be unaware of the clinical significance of early dental symptoms, thereby contributing to delayed diagnoses and treatment. Despite these gaps, few empirical studies have directly quantified the impact of missed nursing care on dental outcomes in low-resource settings. The absence of such care places additional strain on already overburdened healthcare systems, escalating treatment costs and ICU admissions.

This paper presents a policy-oriented perspective on the neglect of preoperative nursing care in dentistry and its public health implications in low-resource environments. Drawing on global examples we examine the structural, educational, and workforce barriers to nursing integration in dental settings. We also offer strategic recommendations to improve early detection, triage, and emergency dental care by strengthening nursing education and promoting interprofessional collaboration. While this discussion is grounded primarily in African contexts, the challenges and policy gaps identified are broadly relevant across other underserved health systems where oral health remains siloed from mainstream medical care.

## Missed nursing care in dentistry and its consequences

The absence of structured nursing roles in dental care represents a significant and underappreciated barrier to effective preoperative assessment and timely intervention across many low and middle-income countries (LMICs). This narrow professional siloing persists despite a growing body of global evidence suggesting that multidisciplinary approaches, especially those involving nursing personnel, improve the early detection and management of oral health emergencies (10). The shortage of trained nursing personnel with dental competencies intensifies this challenge. Across much of sub-Saharan Africa, national nursing curricula often lack dedicated modules on oral health assessment, and dental nurses are rarely employed at the primary care level (11, 12). In India, although the burden of untreated dental infections is high in rural areas, the integration of community health nurses into oral health screening has not been formally adopted (13). Similarly, in Brazil's rural Amazonian regions, nurse-led outreach programs have struggled to incorporate dental triage due to policy and training gaps (14). These examples show a systemic disconnection between general nursing practice and dental health systems in LMICs. The absence of nursing-led screening or triage often leads to missed early signs of systemic deterioration in dental patients, such as trismus, dysphagia, facial cellulitis, or respiratory compromise, delaying interventions that could prevent serious outcomes like sepsis or airway obstruction (15). In many rural clinics across Africa, Southeast Asia, and the Pacific Islands, nurses are often the only available healthcare professionals, yet they lack protocols and training to evaluate or refer patients with dental-origin infections (16). Beyond preoperative triage, the broader public health role of nurses in oral care has been well demonstrated in higher-resource settings. For example, in the United Kingdom and Sweden, nurses in maternal and child health clinics routinely provide anticipatory guidance on early childhood caries (ECC), including bottle-feeding practices, oral hygiene, and dietary counseling (17, 18). In contrast, in many LMICs, such as Nigeria, oral health promotion is rarely integrated into maternal-child health programming, despite evidence that frontline nurses are trusted providers who could effectively deliver these messages (19).

From a systems-level perspective, the consequences of missed preoperative nursing care in dentistry extend beyond individual outcomes. Delays in recognizing and managing odontogenic infections contribute to longer hospital stays, higher inpatient costs, and increased use of intensive care services, resources that are already in short supply (20). In countries like Uganda, where surgical capacity and ICU beds are extremely limited, preventable dental emergencies can destabilize already fragile hospital systems (3, 21).

## Policy implications and recommendations

Addressing the widespread gaps in preoperative nursing care in dentistry requires deliberate, system-wide reforms that elevate oral health as a critical component of patient safety and universal health coverage. A foundational step in addressing these challenges is the integration of oral health into general nursing education. In many LMICs, nurses graduate with little to no formal training in oral health assessment, despite well-established links between dental disease and systemic conditions such as cardiovascular disease, diabetes, and respiratory infections (22). Embedding structured oral health modules within nursing training, similar to pilot programs in Iran, can equip future nurses with the skills to recognize early warning signs of dental emergencies (23, 24). Collaboration among nursing councils, dental schools, and ministries of health is essential to standardize these curricula and ensure they are adapted to local epidemiological realities and resource constraints.

Expanding the role of nurses through task-sharing models is another pragmatic strategy, particularly in rural and underserved regions. The shortage of trained dental professionals is a persistent barrier in many countries. However, evidence from South Africa suggests that nurses, when adequately trained and supported, can successfully conduct basic dental assessments, manage pain, deliver preventive counseling, and triage cases for referral (25). The development of standardized nursing protocols for dental emergencies is also critical. Unlike other areas of medicine where nurses rely on evidence-based guidelines to manage acute conditions, dental care often lacks such structured protocols for nursing assessment or intervention (8, 26). This results in inconsistent care delivery and missed opportunities for early action. National health agencies should prioritize the creation and dissemination of practical, evidence-informed protocols that define nursing responsibilities in managing common dental emergencies, including odontogenic infections, dental trauma, and sepsis of oral origin. These protocols should be integrated into broader triage systems used in primary care clinics and emergency departments, ensuring nurses can confidently assess symptoms, initiate basic interventions, and escalate care when needed. Nurses working in emergency departments, pediatric units, and intensive care settings should be trained to identify oral health complications that may present as systemic illness, such as septicemia or airway compromise due to dental infections, and coordinate care with dental professionals (27). Conversely, dental teams should be included in interprofessional care planning and routine communication within hospitals. Cross-training programs, shared electronic health records, and collaborative referral systems can help streamline care delivery and improve patient outcomes across disciplines.

Finally, systemic change must be supported by national policy and aligned with broader health system goals. Oral health strategies should explicitly recognize the role of nurses not only in education and prevention, but in preoperative assessment, risk stratification, and emergency response. These roles should be integrated into workforce planning, quality improvement frameworks, and universal health coverage policies. Countries like Australia have begun incorporating nurse-led dental screening in Indigenous health services (28), while others, such as Tanzania, are exploring community-based oral health surveillance through primary care teams (29).

## Conclusion

The persistent exclusion of nursing roles from dental care represents a significant gap in global health systems, particularly in resource-limited settings where preventable dental conditions often progress into severe and life-threatening complications. Oral health is inextricably linked to overall well-being, and its neglect within broader healthcare frameworks has implications not only for individual morbidity but also for systemic health outcomes, healthcare costs, and the functionality of primary and emergency care systems. The absence of structured, preoperative nursing involvement in dentistry contributes to delays in the recognition and management of high-risk conditions, particularly in communities with limited access to specialist care. This gap disproportionately affects vulnerable populations, including children, the elderly, and immunocompromised individuals, who are more likely to suffer from delayed diagnosis, inadequate triage, and suboptimal care.

## Data Availability

The original contributions presented in the study are included in the article/Supplementary Material, further inquiries can be directed to the corresponding author.

## References

[1] Foláyan MO Bhayat A Mikhail SS Ndembi N El Tantawi M. Resources for oral health in Africa. Front Oral Health. (2025) 6:1540944. 10.3389/froh.2025.1540944 39989602 PMC11842384

[2] The Challenge of Oral Disease—A call for global action. The Oral Health Atlas. 2nd Eds Geneva: FDI World Dental Federation (2015).

[3] Foláyan MO Ishola AG Bhayat A El Tantawi M Ndembi N. Strengthening health systems to tackle oral diseases in Africa: Africa centers for disease control and prevention’s role. Front Public Health. (2025) 13:1539805. 10.3389/fpubh.2025.1539805 39916711 PMC11798923

[4] Edfeldt K Nyholm L Jangland E Gunnarsson AK Fröjd C Hauffman A. Missed nursing care in surgical care- a hazard to patient safety: a quantitative study within the inCHARGE programme. BMC Nurs. (2024) 23(1):233. 10.1186/s12912-024-01877-1 38584285 PMC10999080

[5] Andersson I Eklund AJ Nilsson J Bååth C. Prevalence, type, and reasons for missed nursing care in municipality health care in Sweden—a cross sectional study. BMC Nurs. (2022) 21(1):95. 10.1186/s12912-022-00874-6 35462537 PMC9035238

[6] Adekunle AA Uti OG Sofola OO. Correlates of illness behaviour related to orofacial infections of odontogenic origin among adults in a semi urban community in Nigeria. Ghana Med J. (2019) 53(4):294–8. 10.4314/gmj.v53i4.7 32116341 PMC7036443

[7] Mathew GC Ranganathan LK Gandhi S Jacob ME Singh I Solanki M Odontogenic maxillofacial space infections at a tertiary care center in north India: a five-year retrospective study. Int J Infect Dis. (2012) 16(4):e296–302. 10.1016/j.ijid.2011.12.014 22365137

[8] Dagnew ZA Abraham IA Beraki GG Mittler S Achila OO Tesfamariam EH. Do nurses have barriers to quality oral care practice at a generalized hospital care in Asmara, Eritrea? A cross-sectional study. BMC Oral Health. (2020) 20(1):149. 10.1186/s12903-020-01138-y 32434570 PMC7240980

[9] Dagnew ZA Abraham IA Beraki GG Tesfamariam EH Mittler S Tesfamichael YZ. Nurses’ attitude towards oral care and their practicing level for hospitalized patients in orotta national referral hospital, Asmara-Eritrea: a cross-sectional study. BMC Nurs. (2020) 19:63. 10.1186/s12912-020-00457-3 32665767 PMC7348104

[10] Nesengani TV Downing C Ten Ham-Baloyi W. Barriers to effective patient care as experienced by nurses in primary healthcare clinics in African countries: a systematic review of qualitative studies. BMC Nurs. (2025) 24(1):232. 10.1186/s12912-025-02877-5 40022067 PMC11869662

[11] Kaguru G Ayah R Mutave R Mugambi C. Integrating oral health into primary health care: a systematic review of oral health training in sub-Saharan Africa. J Multidiscip Healthc. (2022) 15:1361–7. 10.2147/JMDH.S357863 35761842 PMC9233489

[12] Gallagher JE Mattos Savage GC Crummey SC Sabbah W Varenne B Makino Y. Oral health workforce in Africa: a scarce resource. Int J Environ Res Public Health. (2023) 20(3):2328. 10.3390/ijerph20032328 36767693 PMC9915704

[13] Batra P Saini P Yadav V. Oral health concerns in India. J Oral Biol Craniofac Res. (2020) 10(2):171–4. 10.1016/j.jobcr.2020.04.011 32489817 PMC7254460

[14] Nunes FGDS Santos AMD Carneiro ÂO Fausto MCR Cabral LMDS Almeida PF. Challenges to the provision of specialized care in remote rural municipalities in Brazil. BMC Health Serv Res. (2022) 22(1):1386. 10.1186/s12913-022-08805-6 36419054 PMC9682659

[15] Li X Yao L Yang X Huang M Zhang B Yu T Perceptions, barriers, and challenges of oral care among nursing assistants in the intensive care unit: a qualitative study. BMC Oral Health. (2024) 24:235. 10.1186/s12903-024-03979-3 38355476 PMC10868102

[16] McCullough K Bayes S Whitehead L Williams A Cope V. Nursing in a different world: remote area nursing as a specialist-generalist practice area. Aust J Rural Health. (2022) 30(5):570–81. 10.1111/ajr.12899 35770878 PMC9796301

[17] Saikia A Aarthi J Muthu MS Patil SS Anthonappa RP Walia T Sustainable development goals and ending ECC as a public health crisis. Front Public Health. (2022) 10:931243. 10.3389/fpubh.2022.931243 36330110 PMC9624450

[18] Anil S Anand PS. Early childhood caries: prevalence, risk factors, and prevention. Front Pediatr. (2017) 5:157. 10.3389/fped.2017.00157 28770188 PMC5514393

[19] Adeniyi A Akama G Lukandu O Ikemeri JE Jumah A Chelagat S Reducing maternal and child oral health disparities in sub-Saharan Africa through a community-based strategy. Front Oral Health. (2024) 5:1429332. 10.3389/froh.2024.1429332 39005710 PMC11239421

[20] Janatolmakan M Khatony A. Explaining the consequences of missed nursing care from the perspective of nurses: a qualitative descriptive study in Iran. BMC Nurs. (2022) 21(1):59. 10.1186/s12912-022-00839-9 35287687 PMC8918588

[21] Anesi GL Kerlin MP. The impact of resource limitations on care delivery and outcomes: routine variation, the coronavirus disease 2019 pandemic, and persistent shortage. Curr Opin Crit Care. (2021) 27(5):513–9. 10.1097/MCC.0000000000000859 34267075 PMC8416747

[22] Palmer SJ. Improving oral health outcomes through community nursing. Br J Community Nurs. (2025) 30(5):228–31. 10.12968/bjcn.2025.0058 40315165

[23] Tabatabaei SH Owlia F Ayatollahi F Maybodi FR Ahadian H Azizian F Nurses’ educational needs in the oral health of inpatients at Yazd province in Iran: a delphi study. BMC Nurs. (2020) 19(1):120. 10.1186/s12912-020-00517-8 33308231 PMC7733290

[24] Bhagat V Hoang H Crocombe LA Goldberg LR. Incorporating oral health care education in undergraduate nursing curricula—a systematic review. BMC Nurs. (2020) 19:66. 10.1186/s12912-020-00454-6 32684840 PMC7359291

[25] Thema LK Singh S. Integrated primary oral health services in South Africa: the role of the PHC nurse in providing oral health examination and education. Afr J Prim Health Care Fam Med. (2013) 5(1):413. 10.4102/phcfm.v5i1.413

[26] Tsui PK Chau PH Wong JYH Wang MP Gao X Lam OLT Oral care knowledge, attitude and practice among nursing staff in acute hospital settings in Hong Kong. PLoS One. (2023) 18(8):e0289953. 10.1371/journal.pone.0289953 37582111 PMC10427009

[27] Abdelhafez AI Tolba AA. Nurses’ practices and obstacles to oral care quality in intensive care units in upper Egypt. Nurs Crit Care. (2023) 28(3):411–8. 10.1111/nicc.12736 34855285

[28] Poirier B Sethi S Hedges J Jamieson L. Building an understanding of indigenous health Workers’ role in oral health: a qualitative systematic review. Community Dent Oral Epidemiol. (2023) 51(2):169–79. 10.1111/cdoe.12743 35324023

[29] Gudsoorkar P Nolan R Kafle S Dubey A. Exploration of oral hygiene practices, oral health status, and related quality of life of individuals residing in the Rorya district of Tanzania, East Africa. Front Oral Health. (2024) 5:1435555. 10.3389/froh.2024.1435555 39411580 PMC11473497

